# Diffuse myocardial fibrosis in hypertrophic cardiomyopathy can be identified by cardiovascular magnetic resonance, and is associated with left ventricular diastolic dysfunction

**DOI:** 10.1186/1532-429X-14-76

**Published:** 2012-10-29

**Authors:** Andris H Ellims, Leah M Iles, Liang-han Ling, James L Hare, David M Kaye, Andrew J Taylor

**Affiliations:** 1 Heart Centre, Alfred Hospital, Melbourne, Australia; 2 Baker IDI Heart and Diabetes Research Institute, Melbourne, Australia

**Keywords:** Hypertrophic cardiomyopathy, Magnetic resonance imaging, Myocardial fibrosis, T_1_ mapping

## Abstract

**Background:**

The presence of myocardial fibrosis is associated with worse clinical outcomes in hypertrophic cardiomyopathy (HCM). Cardiovascular magnetic resonance (CMR) with late gadolinium enhancement (LGE) sequences can detect regional, but not diffuse myocardial fibrosis. Post-contrast T_1_ mapping is an emerging CMR technique that may enable the non-invasive evaluation of diffuse myocardial fibrosis in HCM. The purpose of this study was to non-invasively detect and quantify diffuse myocardial fibrosis in HCM with CMR and examine its relationship to diastolic performance.

**Methods:**

We performed CMR on 76 patients - 51 with asymmetric septal hypertrophy due to HCM and 25 healthy controls. Left ventricular (LV) morphology, function and distribution of regional myocardial fibrosis were evaluated with cine imaging and LGE. A CMR T_1_ mapping sequence determined the post-contrast myocardial T_1_ time as an index of diffuse myocardial fibrosis. Diastolic function was assessed by transthoracic echocardiography.

**Results:**

Regional myocardial fibrosis was observed in 84% of the HCM group. Post-contrast myocardial T_1_ time was significantly shorter in patients with HCM compared to controls, consistent with diffuse myocardial fibrosis (498 ± 80 ms vs. 561 ± 47 ms, p < 0.001). In HCM patients, post-contrast myocardial T_1_ time correlated with mean E/e’ (r = −0.48, p < 0.001).

**Conclusions:**

Patients with HCM have shorter post-contrast myocardial T_1_ times, consistent with diffuse myocardial fibrosis, which correlate with estimated LV filling pressure, suggesting a mechanistic link between diffuse myocardial fibrosis and abnormal diastolic function in HCM.

## Background

Hypertrophic cardiomyopathy (HCM) is a relatively common inherited cardiac disease defined by the presence of otherwise unexplained left ventricular (LV) hypertrophy associated with non-dilated ventricular chambers [1,2]. Inheritance is autosomal dominant and mutations generally involve sarcomeric genes [3,4]. Patients may develop symptoms from LV outflow tract (LVOT) obstruction [2] and, in the presence of certain high-risk features, an implantable cardioverter defibrillator is recommended to reduce the risk of sudden cardiac death [5]. Many patients, however, develop symptoms of breathlessness due to diastolic dysfunction which is largely independent of the severity of LVOT obstruction [6]. As patients with HCM are known to develop diffuse, as well as regional myocardial fibrosis, this may represent a mechanism for increased myocardial stiffness leading to impaired diastolic filling [7].

Cardiovascular magnetic resonance (CMR) after intravenous administration of gadolinium contrast can non-invasively characterize myocardial tissue [8]. Late gadolinium enhancement (LGE) in HCM patients represents replacement fibrosis histologically [9,10] and portends a worse outcome [11]. LGE has been noted in up to 80% of HCM patients [12-14], typically within the thickened interventricular septum or at points of insertion of the RV free wall [15,16]. Histologic evaluation of myocardial tissue in HCM, however, has demonstrated a more global, or diffuse, increase in fibrosis that cannot be detected by standard CMR LGE sequences [7,10].

Post-contrast myocardial longitudinal relaxation time (T_1_) mapping is an emerging CMR technique that can detect and quantify diffuse interstitial myocardial fibrosis [17] without the necessity for invasive biopsy. Different T_1_ mapping protocols have identified diffuse myocardial fibrosis in several cardiac disease states [8,18], however research involving HCM is limited [19,20].

A comprehensive non-invasive evaluation of regional and diffuse myocardial fibrosis in a typical cohort of HCM patients has not previously been described. This study was undertaken to detect and quantify diffuse myocardial fibrosis in these patients using a histologically-validated CMR post-contrast myocardial T_1_ mapping technique [17]. Furthermore, we investigated the relationships of both patterns of fibrosis to LV diastolic performance and clinical manifestations.

## Methods

### Patient selection

All research was performed at the Alfred Hospital, Melbourne, Australia between August 2010 and October 2011. Fifty-one consecutive patients (39 men, 12 women) referred to our CMR department for the further evaluation of asymmetric septal hypertrophy (ASH) due to HCM were invited to participate. ASH was defined as an interventricular septum thickness of ≥15 mm with a ratio of septal-to-lateral ventricular wall thickness of ≥1.3:1.0 as measured by echocardiography, and the diagnosis of HCM required the absence of another condition that could cause the degree of hypertrophy observed [1]. Twenty-five asymptomatic subjects with no documented history of cardiovascular disease formed a healthy control group.

Exclusion criteria included previous septal reduction therapy; previously documented coronary artery disease or current symptoms suggestive of coronary artery disease; atrial fibrillation; diabetes mellitus; contraindications to CMR, including pacemaker and defibrillator implantation; and significant renal dysfunction (estimated glomerular filtration rate (eGFR) <30 mL/min/1.73m^2^).

Informed consent was obtained from all participants and the study was conducted in accordance with the Alfred Hospital Ethics Committee’s guidelines.

### CMR protocol

#### CMR sequences

We performed CMR using a clinical 1.5-T scanner (Signa HD 1.5-T, GE Healthcare, Waukesha, Wisconsin, USA). All sequences were acquired during a breath-hold of 10–15 s. LV function was assessed by a steady-state free precession (SSFP) pulse sequence (repetition time [TR] = 3.8 ms, echo time [TE] = 1.6 ms, 30 phases, slice thickness 8 mm).

Initial cine CMR sequences were performed in 3 standard long-axis (4-, 3- and 2-chamber views) and short-axis slices (basal, mid, and apical), kept identical for each subsequent sequence throughout the CMR examination [21]. From an end-diastolic, 4-chamber, long-axis view, 5 equally spaced short-axis slices were planned, so that the 2 outer slices lined up exactly either with the tip of the apex or the mitral annulus. The 2 outer slices were then deleted, leaving 3 slices corresponding to typical basal, mid, and apical short-axis views. To calculate LV volume and function, a contiguous short-axis SSFP stack was acquired (8 mm slice thickness, no gap), extending from the mitral valve annulus to the LV apex.

LGE was evaluated 10 min after a bolus of gadolinium-diethylene triamine penta-acetic acid (DTPA) (0.2 mmol/kg BW Magnevist, Schering, Germany) to identify regional fibrosis using a T_1_-weighted inversion recovery gradient echo technique (TR 7.1 ms, TE 3.1 ms, inversion time [TI] individually determined to null the myocardial signal, slice thickness 8 mm, matrix 256 × 192, number of acquisitions = 2). The TI optimization sequence was performed 8 min post-gadolinium administration and was a fast gradient echo, inversion recovery, gated, multi-phase acquisition, commencing at an inversion time of 150 ms and increasing in 25 ms increments to 250 ms, in a single mid-ventricular short-axis slice. A visual determination of the optimum TI to null the myocardial signal was then made. LGE imaging was performed using both standard long-axis and short-axis views of the LV (including a contiguous stack of slices from the mitral valve annulus to the apex).

For the evaluation of diffuse myocardial fibrosis, a T_1_ mapping sequence was used to cycle through acquisition of images obtained at the 3 standard short-axis levels over a range of inversion times [17]. The sequence consisted of an electrocardiogram-triggered, inversion-recovery prepared, 2-dimensional fast gradient echo sequence employing variable temporal sampling of -space (VAST) [22] (Global Applied Science Laboratory, GE Healthcare). Ten images were acquired sequentially at increasing inversion times (75 to 750 ms), commencing 20 min after the bolus of gadolinium-DTPA and over a series of 3 to 5 breath-holds. Imaging parameters were TR/TE: 3.7 ms/1.2 ms, flip angle: 20°, 256×128 acquisition matrix, 36 × 27 cm field of view, slice thickness 8 mm, TI: 75–750 ms, trigger delay 300 ms, and views per segment = 24. The acquisition of all ten images for each myocardial slice was completed in approximately three minutes. These images were then processed with a curve fitting technique to generate T_1_ maps.

#### Evaluation of LV dimensions, function, mass and regional fibrosis

Volumetric analysis of the LV was performed using the summation of disc method. Regional fibrosis was identified by LGE within the myocardium, defined quantitatively by a myocardial post-contrast signal intensity 6 SD above that within a reference region of remote myocardium (without LGE) within the same slice [23]. LGE was defined as being present only if it was identified in two orthogonal views.

#### Evaluation of diffuse fibrosis with T_1_ mapping

**Figure 1:**
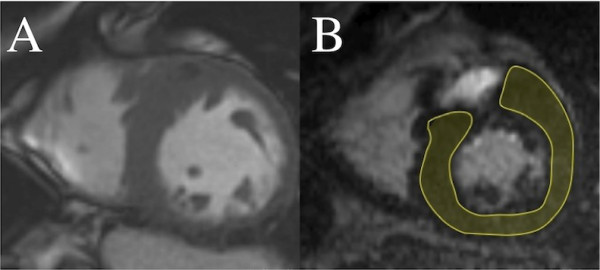
**Calculation of post-contrast myocardial T**_**1**_**time in HCM.** (**A**) Short-axis steady-state free precession image of a patient with asymmetric septal hypertrophy due to HCM. (**B**) Post-contrast T_1_ mapping image at the same short-axis level, with the region of interest (shaded) chosen to include LV myocardium, but exclude a region of late gadolinium enhancement

Following image acquisition, the ten short-axis images of varying inversion times were transferred to an external computer for analysis using a dedicated research software package (Cinetool, Global Applied Science Laboratory, GE Healthcare). This provided the ability to analyze regions of interest (ROIs) to find average T_1_ for that area, as well as a pixel-by-pixel determination of T_1_, by fitting data acquired at various preparation times to the exponential curve: M_z_ (t = TI) = M_0_(A – B[e^-t/T1^=]), relating the sample magnetization M_z_ observed at the time t = TI to the equilibrium magnetization M_0_ and sample T_1_, where TI denotes inversion time for an inversion recovery experiment. For each short-axis image, a ROI was drawn around the entire LV myocardium (excluding papillary muscles) to calculate post-contrast myocardial T_1_ time. In subjects with regional fibrosis detected by LGE, these regions were excluded from the ROI for the primary analysis of post-contrast myocardial T_1_ time (see Figure 1). To investigate for regional variations in T_1_ time, separate ROIs were drawn around hypertrophied (defined as CMR-measured wall thickness ≥ 11 mm during diastole) and non-hypertrophied LV myocardium for each short-axis slice.

#### Evaluation of diastolic function

Transthoracic echocardiography with a standard clinical protocol was performed on all patients immediately prior to CMR. Diastolic function was assessed by a combination of mitral inflow pattern (E to A ratio and deceleration time) and mitral annular velocities (e’, measured at the septal and lateral aspects of the mitral annulus in the apical 4-chamber view). Additionally, mitral E/e’ (septal, lateral and mean) was chosen as an index of LV filling pressure.

#### Image analysis

All CMR and echocardiogram images were interpreted by two experienced readers unaware of the subjects’ clinical information and the results of other diagnostic tests. Endocardial and epicardial LV contours were drawn manually for each diastolic and systolic frame, excluding papillary muscles.

#### Statistical methods

All data are expressed as mean ± SD unless otherwise indicated. Comparison of continuous variables utilized unpaired Student t-test. Comparisons of proportions were made with chi-squared analysis. Multiple comparisons were performed by analysis of variance (ANOVA), with post hoc testing (Holm-Sidak method) as appropriate. Correlations of variables were determined by calculating the Pearson Product Moment. Multiple linear regression was used to determine the independence of correlations observed on simple linear regression, with all correlations with a p value < 0.1 entered into multiple linear regression analysis. Binary categorical variables were entered into the analyses using dummy coding. Linear regression and Bland-Altman analysis were performed to assess interobserver agreement. For all comparisons, a p value of < 0.05 was considered significant, and all reported p values are 2-tailed. All analyses were conducted using Stata software version 11.1 (StataCorp, College Station, Texas).

## Results

### Clinical and demographic data

A total of 76 patients were evaluated during the study period, comprising 51 patients with ASH due to HCM and 25 control subjects. Baseline characteristics of both groups are presented in Table 1. Patients in both groups were of a similar age. 76% of HCM patients were male, compared to 72% of control subjects. Body mass index (BMI) was significantly higher in the HCM group (27.5 ± 4.9 kg/m^2^ vs. 24.1 ± 2.7 kg/m^2^, p < 0.001). One-third of HCM patients had a first-degree relative previously diagnosed with HCM. 74% of patients reported symptoms attributable to HCM (including chest pain, dyspnoea, presyncope and/or syncope). The severity of dyspnoea in HCM patients was generally mild, with no patients experiencing New York Heart Association (NYHA) class III/IV symptoms. Three-quarters of the HCM group were receiving beta-blocker and/or non-dihydropyridine calcium channel blocker therapy. There were no significant differences in heart rate, systolic blood pressure, hematocrit, or renal function between the HCM and control groups.

**Table 1:** Baseline characteristics

	**HCM (n=51)**	**Control (n=25)**	**p value**
Age, y	48 ± 14	48 ± 17	0.8
Males, n (%)	39 (76%)	18 (72%)	0.7
Body mass index, kg/m^2^	27.5 ± 4.9	24.1 ± 2.7	<0.001
Family history of HCM, n (%)	17 (33%)	-	
Symptoms, n (%)			
Chest pain	16 (31%)	-	
Dyspnoea	28 (55%)	-	
NYHA class I or II	51 (100%)	-	
Presyncope	18 (35%)	-	
Syncope	6 (12%)	-	
Medications, n (%)			
Beta-blocker	29 (57%)	-	
Calcium channel blocker	10 (20%)	-	
Resting heart rate, beats/min	60 ± 10	62 ± 9	0.6
Systolic blood pressure, mm Hg	129 ± 16	133 ± 18	0.4
Hematocrit	0.43 ± 0.04	0.42 ± 0.03	0.3
eGFR, ml/min/1.73 m^2^	81 ± 12	86 ± 8	0.14

NYHA indicates New York Heart Association; and eGFR, estimated glomerular filtration rate.

### CMR findings

CMR was successfully completed in all 76 patients and the results are displayed in Table 2. The HCM group had a significantly higher LV ejection fraction and a greater LV mass indexed to body surface area (BSA) compared to the control group. LV end-diastolic volumes indexed to BSA were similar in both groups. The maximum ventricular septal thickness of HCM patients was 20 ± 3 mm compared to 8 ± 2 mm for control subjects, while the ratio of septal-to-lateral ventricular wall thickness for the HCM group was 2.3:1. LGE was observed in 84% of HCM patients, generally localized to the ventricular septum or points of RV free wall insertion. Subendocardially-based LGE, consistent with ischemic scar, was not observed in any patient. The mean quantity of LGE, expressed as a percentage of LV mass, was 6.1 ± 7.7%.

**Table 2:** Cardiac MRI data

	**HCM (n=51)**	**Control (n=25)**	**p value**
LVEDV, ml	162 ± 36	156 ± 35	0.5
LVEDV indexed, ml/BSA	81 ± 14	83 ± 13	0.5
LVESV, ml	50 ± 17	63 ± 18	<0.01
LV stroke volume, ml	112 ± 27	94 ± 20	<0.01
LVEF, %	70 ± 7	60 ± 6	<0.001
LV mass, g	178 ± 54	98 ± 25	<0.001
LV mass indexed, g/BSA	89 ± 25	52 ± 9	<0.001
Septal thickness, mm	20 ± 3	8 ± 2	<0.001
Lateral wall thickness, mm	9 ± 2	8 ± 1	<0.05
Septal:lateral wall thickness	2.3 ± 0.7	1.1 ± 0.9	<0.001
Presence of LGE, n (%)	43 (84%)	0 (0%)	
Quantity of LGE, % of LV mass	6.1 ± 7.7	0	

LVEDV indicates left ventricular end-diastolic volume; LVEF, left ventricular ejection fraction; LVESV, left ventricular end-systolic volume; and LGE, late gadolinium enhancement.

### Post-contrast myocardial T_1_ time in HCM and control subject

The timing of acquisition of T_1_ mapping sequences after the delivery of the gadolinium contrast bolus was similar in both groups (23:43 ± 3:57 min vs. 22:34 ± 3:48 min, p = 0.2). There was an excellent correlation between the two blinded CMR specialist reviewers when they independently calculated myocardial T_1_ times (r = 0.99, p < 0.001). Bland-Altman analysis showed good inter-observer agreement (mean difference in T_1_ time was 0.29 ± 19.28 ms, limits of agreement were −38.29 to 38.84). Patients with HCM had significantly shorter post-contrast myocardial T_1_ times compared with controls (498 ± 80 ms vs. 561 ± 47 ms, p < 0.0001) (Table 3). When regions of LGE were included in the analysis of HCM patients, a further reduction in T_1_ time (483 ± 85 ms) was observed. Post-contrast T_1_ times were similar in both hypertrophied and non-hypertrophied LV myocardium (503 ± 127 ms vs. 497 ± 111 ms, p = 0.7). There was no difference in post-contrast T_1_ times of the LV blood pool between the HCM and control groups (304 ± 31 ms vs. 306 ± 22 ms respectively, p = 0.8).

**Table 3:** **Post-contrast myocardial T**_**1**_**mapping data**

	**HCM (n=51)**	**Control (n=25)**	**p value**
Post-contrast T_1_ time, ms			
LV myocardium, excluding LGE	498 ± 80	561 ± 47	<0.0001
LV myocardium, including LGE	483 ± 85	561 ± 47	<0.0001
LV blood pool	304 ± 31	306 ± 22	0.8

LGE indicates late gadolinium enhancement.

### Echocardiography findings

Echocardiographic data are presented in Table 4. Left atrial volume indexed to BSA was higher in the HCM group. The mean resting LVOT gradient in HCM patients was 26 ± 36 mm Hg. Septal, lateral, and mean early diastolic mitral annular velocities (e’) as measured by tissue Doppler imaging (TDI) were all lower in the HCM group compared to controls, and septal, lateral, and mean E/e’ were higher in the HCM group.

**Table 4:** Echocardiography data

	**HCM (n=51)**	**Control (n=25)**	**p value**
Left atrial volume indexed, ml/m^2^	51 ± 18	32 ± 10	<0.001
Resting LVOT gradient, mm Hg	26 ± 36	4 ± 1	<0.001
Mitral E velocity, cm/s	0.8 ± 0.2	0.7 ± 0.2	0.1
Mitral A velocity, cm/s	0.6 ± 0.3	0.6 ± 0.2	0.5
E/A ratio	1.4 ± 0.6	1.4 ± 0.5	0.7
Deceleration time, ms	217 ± 51	188 ± 36	<0.01
Septal e’, cm/s	6.0 ± 1.7	8.8 ± 3.0	<0.001
Lateral e’, cm/s	8.3 ± 2.6	12.0 ± 4.0	<0.001
Mean e’, cm/s	7.1 ± 1.9	10.4 ± 3.3	<0.001
Septal E/e’ ratio	14.4 ± 5.8	8.9 ± 3.2	<0.001
Lateral E/e’ ratio	10.6 ± 4.7	6.4 ± 1.9	<0.001
Mean E/e’ ratio	12.5 ± 4.9	7.7 ± 2.4	<0.001

LVOT indicates left ventricular outflow tract.

### Correlates of post-contrast myocardial T_1_ time in HCM patients

Significant negative correlations were observed between post-contrast myocardial T_1_ time and age and BMI (Table 5). Following multiple linear regression analysis, the correlation between T_1_ time and age remained significant. There were no associations between T_1_ time and the presence of symptoms or NYHA class. There were no significant correlations between T_1_ time and resting heart rate, blood pressure, hematocrit or eGFR. The presence and quantity of LGE (expressed as a percentage of LV mass) did not correlate with post-contrast myocardial T_1_ time.

**Table 5:** **Predictors of post-contrast myocardial T**_**1**_**time in HCM group by simple and multiple linear regression**

	**Simple linear regression**		**Multiple linear regression**	
	**r**	**p value**	**β**	**p value**
Age	−0.41	<0.01	−0.34	0.02
Male	−0.14	0.3		
Body mass index	−0.35	<0.05	−0.24	0.08
Family history of HCM	0.20	0.2		
Presence of symptoms	−0.17	0.2		
NYHA class	−0.16	0.3		
Resting heart rate	0.13	0.4		
Systolic blood pressure	−0.12	0.4		
Diastolic blood pressure	0.12	0.4		
Hematocrit	−0.29	0.11		
eGFR	0.26	0.3		
Presence of LGE	0.18	0.21		
Quantity of LGE	−0.13	0.39		

NYHA indicates New York Heart Association; eGFR, estimated glomerular filtration rate; and LGE, late gadolinium enhancement.

### Correlates of diastolic dysfunction in HCM patients

**Figure 2:**
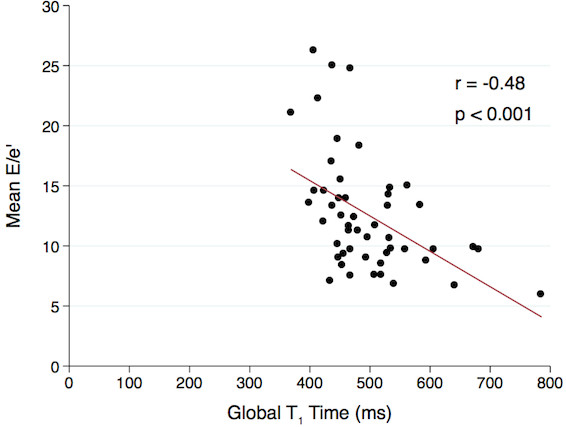
**Post-contrast myocardial T**_**1**_**time and mean E/e’ in HCM patients.** A significant negative correlation was observed between post-contrast myocardial T_1_ time and mean E/e’ (r = −0.48, p < 0.001)

Using mean E/e’ as a measure of diastolic dysfunction, simple linear regression demonstrated significant positive correlations with age, indexed LV mass, maximum septal thickness, indexed left atrial volume, and resting LVOT gradient. A negative correlation was observed between mean E/e’ and post-contrast myocardial T_1_ time (see Figure 2). No significant correlation was observed between the amount of LGE and mean E/e’. In multivariate analysis, the correlation between mean E/e’ and T_1_ time remained significant (Table 6).

**Table 6:** Predictors of mean E/e’ in HCM group by simple and multiple line regression

	**Simple linear regression**		**Multiple linear regression**	
	**r**	**p value**	**β**	**p value**
Baseline characteristics				
Age	0.33	<0.05	0.11	0.4
Body mass index	0.05	0.7		
NYHA class	0.20	0.15		
CMR parameters				
LVEDV indexed	0.23	0.11		
LVEF	−0.10	0.5		
LV mass indexed	0.30	<0.05	−0.10	0.6
Septal thickness	0.28	<0.05	0.31	0.05
Presence of LGE	−0.16	0.3		
Quantity of LGE	0.16	0.3		
Post-contrast myocardial T_1_ time	−0.48	<0.001	−0.34	0.02
Echocardiography parameters				
Left atrial volume indexed	0.38	<0.01	0.24	0.06
Resting LVOT gradient	0.46	<0.001	0.29	0.03

NYHA indicates New York Heart Association; LVEDV, left ventricular end-diastolic volume; LVEF, left ventricular ejection fraction; LGE, late gadolinium enhancement; and LVOT, left ventricular outflow tract.

## Discussion

Using a previously histologically-validated post-contrast T_1_ mapping CMR technique [17], we demonstrated that HCM patients have significantly shorter post-contrast myocardial T_1_ times compared to healthy controls, consistent with the presence of diffuse interstitial myocardial fibrosis. Furthermore, the amount of this fibrosis correlated with echocardiographic measures of LV filling pressure, suggesting a mechanistic link between diffuse fibrosis and abnormal diastolic function in HCM.

Previously, cardiac biopsy was the only means of comprehensively evaluating both regional and diffuse patterns of myocardial fibrosis. However, invasive endomyocardial biopsy is associated with significant procedural risks and the ability to non-invasively image diffuse fibrosis in patients with HCM would be a significant advance. Additionally, biopsies obtained via this technique are mainly derived from the right ventricular portion of the interventricular septum and may not accurately reflect fibrotic processes occurring in either the hypertrophied LV septum or other ventricular segments.

Research utilizing CMR to assess myocardial fibrosis in typical cohorts of HCM patients has previously only identified regional patterns of fibrosis with LGE sequences [11-14,24]. This focus on LGE in HCM has led to improved accuracy in the diagnosis of this condition in patients with unexplained myocardial hypertrophy and may enhance risk stratification for sudden death [25]. Not all patients with HCM, however, exhibit LGE. Our study detected diffuse myocardial fibrosis in HCM patients both with and without LGE and there was no association observed between the amount of LGE and post-contrast myocardial T_1_ time. Additionally, post-contrast T_1_ times of hypertrophied and non-hypertrophied myocardium did not differ, reaffirming the histologically-proven [7] diffuse nature of fibrosis in HCM. In contrast, the presence of LGE correlates with segmental LV wall thickness [15,26]. These findings imply that diffuse and regional myocardial fibrosis in HCM are distinct entities. The relative quantities of these two types of fibrosis varied markedly between HCM patients in our study and may partially account for the range of clinical manifestations in this heterogeneous disease. There was a significant overlap in post-contrast myocardial T_1_ times between HCM patients and control subjects. Possible explanations for this include; variability in the fibrotic content in the myocardium of healthy controls (including age-related changes); subclinical myocardial disease; and the aforementioned heterogeneity of the extent of diffuse myocardial fibrosis in HCM.

The relationship between reduced post-contrast myocardial T_1_ times and diastolic dysfunction has been described in a group of patients with clinical heart failure [17]. Recently, in a cohort of heart failure patients with preserved systolic function, a correlation was noted between the amount of collagen type 1 found on endomyocardial biopsy and echocardiographic indices of diastolic dysfunction [27]. Utilizing early mitral inflow to early diastolic mitral annular velocity (E/e’) as a non-invasive measure of increased LV filling pressure [28-31], our study suggests a mechanistic link between higher LV filling pressures in HCM patients and diffuse myocardial fibrosis. Interestingly, we did not observe a significant correlation between the amount of regional myocardial fibrosis, as detected by LGE, and estimated LV filling pressure.

Animal studies have demonstrated the anti-fibrotic effects of medications that inhibit the angiotensin II system [32]. Therefore, the ability to non-invasively evaluate diffuse fibrosis in HCM is likely to enhance our understanding of pathogenesis and disease progression and may enable therapeutic trials of potential anti-fibrotic agents. Furthermore, as it is uncertain as to when the active pro-fibrotic state in the myocardium of patients with HCM occurs, serial imaging using T_1_ mapping techniques over a patient’s lifetime may glean crucial information about the timing of this process.

### Study limitations

Our research has several limitations. Despite including consecutive patients with asymmetric HCM referred to our CMR centre, no patient experienced class III or IV NYHA symptoms. Further studies involving patients with more severe symptoms, whether due to intra-cavitary obstruction and/or restrictive physiology, would be required to demonstrate whether patients with a greater disease burden might have even lower post-contrast myocardial T_1_ times. Also, variations in the timing of image acquisition after contrast administration as well as heart rate, hematocrit and renal function have been proposed as potential confounders to the interpretation of post-contrast T_1_ times [8]. Various T_1_ mapping techniques have been designed to attempt to address these issues, including an approach that utilized a continuous infusion of contrast to achieve equilibrium [20]. A T_1_ mapping technique to calculate the extracellular volume (ECV) of the myocardium has also been described [33]. Currently, no consensus exists on which is the most accurate CMR T_1_ mapping method, with a number of differing techniques demonstrating significant correlations between post-contrast myocardial T_1_ time and histologically-quantified fibrosis [17,20]. We observed no significant differences in baseline values for these putative confounding factors between our study groups and, after statistical analysis, could not identify any significant correlations with any of these factors and post-contrast myocardial T_1_ times. In addition, post-contrast blood pool T_1_ times were similar in both groups, strongly suggesting that the lower myocardial T_1_ times in HCM patients compared to controls were not due to differences in contrast medium kinetics. Importantly, numerous studies have utilized a similar T_1_ mapping technique to that used in our study, and have demonstrated shortened myocardial post-contrast T_1_ times in humans with a wide range of conditions known to be associated with diffuse myocardial fibrosis, including systolic heart failure [17], the diabetic heart [18,34], chronic valvular heart disease [35] and remote myocardial remodelling post-myocardial infarction [36]. Finally, myocardial edema, identified by CMR, has been described in some patients with HCM and may be due to acute ischaemia [37]. Myocardial edema can affect T_1_ times [38], however, while its presence was not directly assessed in this study, there was no clinical evidence of recent acute myocardial pathology in any study patient.

## Conclusions

Using CMR post-contrast T_1_ mapping, this study has demonstrated that patients with HCM have reduced post-contrast myocardial T_1_ times, consistent with the presence of diffuse interstitial fibrosis. Furthermore, the independent association of post-contrast myocardial T_1_ time with estimated LV filling pressure (E/e’) suggests a mechanistic link between altered myocardial composition and function. The non-invasive detection of diffuse fibrosis, in combination with standard LGE sequences to identify dense regional fibrosis, now allows a comprehensive evaluation of patterns of fibrosis in this condition. Further research utilizing this technique may enhance our understanding of the relationships between HCM genetic mutations, abnormal myocardial structure and function, and risk stratification and may facilitate the future development of disease-modifying therapies.

## Abbreviations

HCM: Hypertrophic cardiomyopathy; LV: Left ventricle/ventricular; LVOT: Left ventricular outflow tract; CMR: Cardiovascular magnetic resonance; LGE: Late gadolinium enhancement; RV: Right ventricle/ventricular; ASH: Asymmetric septal hypertrophy; eGFR: Estimated glomerular filtration rate; SSFP: Steady-state free precession; ROI: Region of interest; BMI: Body mass index; NYHA: New York Heart Association; BSA: Body surface area; TDI: Tissue Doppler imaging.

## Competing interests

The authors declare that they have no competing interests.

## Authors' contributions

AHE conceived of the study, participated in its design and co-ordination, analyzed echocardiogram and CMR results, and drafted the manuscript. LMI developed the T_1_ mapping technique, completed the ethics submission and analyzed CMR results. LL recruited control patients and analyzed CMR results. JLH analyzed CMR results and assisted in statistical analysis. DMK participated in the study’s design and drafted the manuscript. AJT developed the T_1_ mapping technique, participated in the study’s design and co-ordination, and drafted the manuscript. All authors read and approved the final manuscript.
